# Comminuted fractures of the radial head: Favorable outcome after 15–25 years of follow-up in 19 patients

**DOI:** 10.3109/17453671003717815

**Published:** 2010-04-06

**Authors:** Magnus K Karlsson, Pär Herbertsson, Anders Nordqvist, Jack Besjakov, Per Olof Josefsson, Ralph Hasserius

**Affiliations:** Department of Clinical Sciences, Lund University, Department of Orthopaedics; ^1^Radiology, Malmö University Hospital, MalmöSweden

## Abstract

**Background** There have been few reports on the long-term outcome of comminuted radial head fractures in adults.

**Method** 10 women and 9 men with a mean age of 45 (21–65) years when they sustained a comminuted fracture of the radial head were re-evaluated after 15–25 years. 6 patients had been nonoperatively (NO) treated while 13 had had a radial head excision. The uninjured elbow served as a control.

**Results** At follow-up, 11 patients (4 NO patients) rated their fractured elbow as being without deficits, 7 (1 NO) as being slightly impaired, and 1 (NO) as being severely impaired. Range of motion and elbow strength were not impaired, and even though there were more degenerative changes such as cysts, osteophytes, and sclerosis in the injured elbows by radiography, the prevalence of joint space reduction was not higher.

**Interpretation** Most patients with an isolated comminuted fracture of the radial head treated nonoperatively or with a radial head excision report no or only minor long-term complaints.

## Introduction

Radial head and neck fractures are estimated to account for 25–44% of all elbow fractures (Herbertsson 2004). Minor displaced proximal radius fractures are usually reported to have a favorable outcome (Herbertsson 2004), but some reports have suggested that there is an inferior outcome after severely displaced and comminuted fractures in the same region while authors other have opposed this view (Mason 1954, Arner et al. 1957, Radin and Riseborough 1966, Bakalim 1970, Stephen 1981, Mikic and Vukadinovic 1983, Goldberg et al. 1986, Coleman et al. 1987, Ikeda and Oka 2000, Herbertsson et al. 2004a, b). One possible explanation could be that authors mix displaced 2-fragment fractures, comminuted fractures, radial head fractures, radial neck fractures, fractures in adults, and fractures in children in different proportions in the evaluations. This could be erroneous, as an intra-articular radial head fracture may have an inferior outcome compared to an extra-articular radial neck fracture; and comminuted fracture, which is more often associated with a high-energy trauma (Herbertsson 2004), may have an inferior outcome compared to a displaced two-fragment fracture. This is the reason why we believed that comminuted fractures of the radial head have the worst prognosis of all isolated proximal radius head fractures, and why we specifically evaluated the long-term outcome of comminuted fractures of the radial head in adults.

## Patients and methods

We scrutinized the radiographic archives at our hospital where all radiographs had been saved for the years 1969–1979, when 2,965 individuals were registered with an elbow fracture, and found 756 patients with a radial head or neck fracture, 480 (64%) with a Mason type-I fracture, 222 (29 %) with a Mason type-II fracture, 36 (5%) with a Mason type-III fracture, and 18 (2%) with a Mason type-IV fracture (Figure). Of the 258 individuals with a Mason type-II or type-III fracture, 131 were still living in the region. Of these, 124 agreed to attend a follow-up on average 19 (15–25) years after the injury. 24 had been children (≤ 16 years old) when they sustained the fracture, 76 had sustained a Mason type-II fracture, 5 had sustained a Mason type-III fracture of the radial neck (Mason type-IIIb), and 19 had sustained a Mason type-III fracture of the radial head (Mason type-IIIa). It should also be emphasized that the fractures evaluated in this report had previously been included in a publication reporting the outcome of mixed Mason type-II and type-III fractures (Herbertsson et al. 2004a,b).

**Figure:**
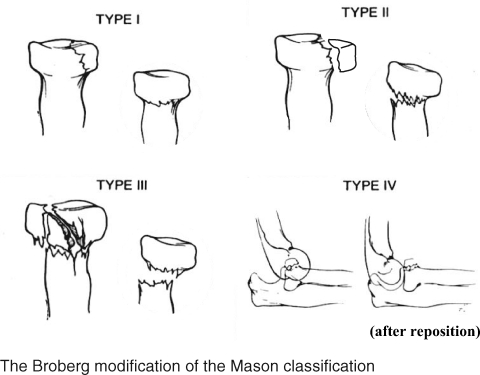
The Broberg modification of the Mason classification

In this paper, we report the outcome in the 19 individuals with a Mason type-IIIa fracture: 10 women and 9 men with a mean age of 45 (21–65) years at injury and with a mean follow-up period of 19 (15–25) years. 5 injuries were the result of high-energy trauma, defined as fall of > 2 meters or a motor-vehicle accident, and 14 injuries were the result of low-energy trauma, defined as a fall of < 2 meters or direct impact. Ten fractures affected the right arm and 9 affected the left. 2 patients were treated with instant mobilization, 6 patients with cast immobilization for 3 weeks, and 11 with a radial head excision within 3 weeks of the injury. 2 of the nonoperatively treated patients had a delayed radial head excision 3 months after the fracture, due to remaining pain. No complications were recorded during or after surgery.

The subjective outcome was assessed in all 19 patients using a questionnaire that evaluated activities of daily living (ADL), elbow pain on loading and at rest, tenderness, range of motion, stability, and strength in the affected elbow. Strength, numbness, and sensitivity in the wrist and the hand were also evaluated. The uninjured arm served as a control in all comparisons. 17 patients returned for the clinical examination, which was performed by 2 of the authors who had not been involved in the treatment of the patients. The flexion and the extension of the elbow and wrist, the pronation and supination of the forearm, and the angle of the extended elbow were measured with a goniometer. The grip strength of the hand was evaluated with a Martin vigorimeter, and the circumference of the arm and forearm was measured with a tape measure 10 cm distal and proximal to the tip of the olecranon. The uninjured arm served as a control. Any difference in the strength of flexion and extension was estimated by subjective comparison of both elbows. Tinel's test in the cubital tunnel was performed in both elbows. Based on the subjective and objective data, we also calculated the Mayo elbow performance score (0–100); below 60 is rated as a poor outcome, 60–74 as fair, 75–89 as good, and > 90 as excellent.

On the basis of the primary radiographs, the fractures were classified according to Mason (1954) as later modified by Broberg et al. (1986). This classification was done by a radiologist with no knowledge of the treatment or the subjective or clinical outcome of the patients. Follow-up radiographs in the 17 cases who accepted having new radiographs taken included anterior-posterior and lateral projections of the elbow. Subchondral cysts, subchondral sclerosis, and/or osteophytes were defined as degenerative changes, and the number of individuals with more than a 1-mm reduction in the joint space was recorded. Miscellaneous pathological entities, such as nonunion, avascular necrosis, proximal radio-ulnar synostosis, and periarticular ossification were also documented. The uninjured elbows served as controls. Comparisons of values for the 2 arms of the same individual were performed with Student's t-test and chi-squared test, with p < 0.05 indicating a significant difference. The ethics committee at Lund University in Sweden approved the study.

## Results

11 patients rated their formerly fractured elbows as being without any subjective complaints, 7 rated them as being with slight impairment, occasionally experienced weakness and occasional pain at load, and 1 reported severe impairment due to pain at rest, tenderness, and weakness. A resection of the radial head had been done in 7 of the patients without subjective complaints and in 6 with subjective complaints.

The valgus angle was greater in the formerly fractured elbows (Table). No other objective deficits were registered. There were 6 patients who had an extension or supination deficit exceeding 10º, while none had a flexion or pronation deficit exceeding 10º. Tinel's test over the cubital tunnel was positive in 5 of the injured elbows and in 1 of the uninjured elbows (p = 0.07). These results left 1 individual with a Mayo elbow performing score of 80, 1 individual with 95, and 17 individuals with 100. There were no differences in range of motion in elbows or wrists, hand grip strength, or arm circumference between patients who had been treated with a radial head excision and those who had been treated nonoperatively.

**Table:** Arm function in 17 patients with a comminuted fracture of the radial head, a mean of 19 years after the injury. Mean (SD)

	Formerly fractured arm	Unfractured arm
Elbow flexion (°)	139 (7)	140 (7)
Elbow extension (°)	–5 (9)	–3 (8)
Forearm pronation (°)	88 (9)	88 (9)
Forearm supination (°)	79 (9)	85 (9)
Elbow valgus angle (°)	12 (7) a	10 (6)
Wrist flexion (°)	64 (15)	65 (16)
Wrist extension (°)	60 (16)	59 (15)
Circumference upper arm (cm)	29 (3)	29 (4)
Circumference forearm (cm)	26 (3)	26 (4)
Grip strength (kp/cm2)	0.7 (0.5)	0.8 (0.5)
^a^ p < 0.05, comparing the formerly fractured arm with the unfractured arm.		

The formerly injured elbows presented with more degenerative changes than the uninjured elbows, namely cysts in 12 vs. 3, sclerosis in 10 vs. 3, and osteophytes in 10 vs. 3 (all p < 0.05). There was no increased prevalence of reduced joint space in the formerly fractured elbows in comparison with the uninjured elbows, and there were no miscellaneous pathological entities in the formerly injured elbows. Furthermore, there were no differences in the proportion of elbows with subchondral cysts, subchondral sclerosis, osteophytes, and/or joint space reduction between elbows treated with a radial head excision and those that had been nonoperatively treated.

## Discussion

In general, we found a good outcome 2 decades on average after a comminuted fracture of the radial head. However, it must be emphasized that our study only included individuals with an isolated fracture of the radial head and not individuals with associated fractures that are often seen in conjunction with a fracture of the radial head (Coleman et al. 1987, Ikeda and Oka 2000).

There are reports with short-term data that both support and refute our findings. In 11 patients with a comminuted fracture of the radial head who were followed for 1–15 years, 7 of whom were treated with partial or total excision of the radial head and 4 of whom were treated nonoperatively, Arner et al. (1957) reported a good or excellent outcome in 9 cases. Of 13 patients with comminuted fractures of the radial head, treated with radial head excision and followed for up to 9 years, Bakalim (1970) reported good results in 7. However, in 5 of the 6 cases with a poor outcome, the radial head fracture was associated with concurrent elbow injuries. In one series involving 31 patients with a comminuted radial head fracture who were followed for a mean of 2 years, all of whom were treated nonoperatively or with excision of the radial head, satisfactory outcome was reported in 24 of the cases (Radin et al. 1966). Eren et al. (2002) reported a similar outcome in 20 patients with a comminuted radial head fracture who were followed for 2–12 years and treated with a radial head excision. However, Mason type-III and type-IV fractures were included in this evaluation.

The most common complaint among the patients in our study was occasional weakness in the previously injured elbow (7 patients) and occasional pain at load (5 patients). These data support previous studies reporting loss of strength to be a common complaint both after comminuted fractures of the radial head and in patients treated with a radial head excision (Herbertsson 2004, Coleman et al. 1987, Ikeda and Oka. 2000). Reduced range of elbow motion has also been described as a common sequel after comminuted fractures of the radial head (Herbertsson et al. 2004a, b, Coleman et al. 1987, Ikeda and Oka 2000, Mason 1954, Goldberg et al. 1986). In a series of 18 patients with a comminuted fracture of the radial head who were followed for 2 years, all of whom were treated with excision of the radial head, Mason (1954) described a mean loss of extension of 25º and a mean loss of rotation of 30º in the formerly fractured elbow. Our findings speak against this view, perhaps due to the fact that we had a longer follow-up period.

Another complication following excision of the radial head is cubitus valgus. Mikic et al. (1983) followed 60 patients treated with radial head excision after isolated radial head fractures for an average of 6 years, and reported an increasing valgus deformity in the formerly injured elbow, also supported by another study that followed 61 patients for 11–33 years (Herbertsson et al. 2004a). We found the same, a larger valgus angle in the formerly fractured elbows than in the uninjured elbows. This deformity is known to increase the risk of developing ulnar neuropathy, also supported by our findings of a higher prevalence of ulnar irritation in the elbow, as estimated by Tinel's sign.

The injured elbows had degenerative radiographic changes more often than the uninjured elbows. The proportion is similar to that in previous publications that have reported 76% degenerative changes in adult individuals with a radial head fracture of Mason type-II or -III (Mason 1954, Broberg 1986). Similar outcome has been reported in several other studies (Herbertsson et al. 2004a, b).

We conclude that few patients with an isolated Mason type-IIIa fracture of the radial head treated with an excision of the radial head or nonoperatively report any serious long-term complaints.
